# An overview of three biocatalysts of pharmaceutical importance synthesized by microbial cultures

**DOI:** 10.3934/microbiol.2021009

**Published:** 2021-04-27

**Authors:** Divakar Dahiya, Poonam Singh Nigam

**Affiliations:** Biomedical Sciences Research Institute, Ulster University, Coleraine Northern Ireland, UK

**Keywords:** Salicylate hydroxylase, Dihydrofolate reductase, Lipase, Aspirin, Methotrexate

## Abstract

This article includes a general overview of the published research on a topic relevant to biomedical sciences research, pharma-industries and healthcare sector. We have presented a concise information on three enzymes. These biomolecules have been investigated for their biocatalytic activities beneficial in the detection of drugs and their metabolites present in micro-quantities in samples of blood, urine, and other body fluids, such as salicylate hydroxylase, and dihydrofolate reductase. Some enzymes are useful in biotransformation of compounds to convert them in an optically active form, such as lipase. The information presented in this article has been collected from the published studies on their catalytic function, and biosynthesis using selected microorganisms. Several diagnostic assays are currently using enzymes as effective biocatalysts to perform the detection-test. For the marketing and consumer's convenience, pharmaceutical companies have designed biosensors and diagnostic kits by incorporating specific enzymes for rapid tests required in pathology, as well as for the quantification of certain metabolites and chemicals in pathology samples in a shorter time. For such purpose use of enzymes synthesized by selected specific microorganisms is economical.

## 1. Enzymes

Enzymes have been widely studied for their chemistry, reactivity and contribution in biochemistry and biological systems. Enzymes have been studied for their important activity as biocatalysts to synthesize added-value metabolites, as well they are being used as important tool in the diagnostics and therapeutics to improve human health. These important biocatalysts regulate the rate of metabolic reactions that take place within a living cell. Enzymes contribute in a wide range of vital functions in any living cell; therefore, these are important biomolecules for all biological systems, whether in micro or macro organisms [1]. Enzymes have established their applications in many sectors, including use of biocatalysts enzymes for bioresource utilization through bio-remediation [2], medical & pharmaceuticals, textiles, paper and pulp, food and drink, etc. [3]–[6]. In biotechnology, enzymes have been manipulated to improve their quality in experiments to speed up biochemical reactions [1],[7].

Enzymes are involved in all known metabolic pathways and are important tools for regulating conditions in a homeostatic manner. These biocatalysts play significant roles in the metabolism of macromolecules for the survival of all living organisms. Inconsistent and weaker functioning of enzymes might lead to the development of some metabolic disorders. Therefore, in recent years research has been focused on measuring the activity of relevant enzymes at various stages for early detection of health related disorders. Currently in medical and healthcare sector, with increasing health problems and an ageing population in several geographical regions, the detection and treatment of disease at an early stage has become very important. Pharmaceutical companies are continuously researching on new practical and affordable diagnostic systems and portable kits for the detection of several diseases rapidly within a shorter duration [8],[9]. This approach has advantages of a rapid test and smaller volume of samples required in reaction. That could be a better and practical alternative in many cases.

The main disadvantages in the use of conventional analytical diagnosis methods are: first of all the precautions should be taken during the collection of samples by patients at home or by staff at care centers to maintain the sterility and avoid any contamination; secondly, sending required quantity of samples under proper storage conditions to pathology labs, without any spillage during transportation; thirdly the analytical methods of testing must be performed in a properly equipped laboratory setting. That requires the availability of qualified skilled biomedical scientists and pathologists to perform these lab-based analytical testing using conventional methods. There are several important enzymes, which could be used for different assays, have been studied for their biosynthesis employing selective microorganisms [10]–[12].

### 1.1. Enzymes from microbial sources

The specific enzymes can be synthesized using purposely selected specific microbial strains in laboratory. They act as biocatalysts to perform reactions in bioprocesses in an economical and faster way, compared to chemically-catalysed reactions. For specific applications, the different functionality of biocatalysts are required, which includes tolerance to a varied range of pH, stability of enzyme activity over a range of temperature and other reaction conditions [1],[3]. Such enzymes have proven their utility in different situations and requirements. Therefore, several enzymes have been isolated, identified and characterized for a number of specific properties, and exploited for industrial purposes [1]–[6].

Based on their applicability, certain microbial enzymes are categorized as thermophilic, acidophilic or alkalophilic. Each category of enzymes has a specific function but thermophilic enzymes have varied applications. Microorganisms producing thermostable enzymes that can perform reaction at higher temperatures would decrease the possibility of microbial contamination at industrial scale processing. In addition, the higher reaction temperature promotes the breakdown of raw materials. Furthermore, the thermotolerant nature of enzymes also helps in enhancing the mass-transfer and reduction of the substrate viscosity during the progress of hydrolysis of substrates. Thermophilic xylanase, amylases and proteases are considered to be of commercial interest [4]. Microbial-synthesized enzymes, including protease, dihydrofolate reductase, lipase, esterase, salicylate hydroxylase and xylanase, have been investigated for their biosynthesis and application, but not all are produced by thermophilic microorganisms.

In this article, we have included the published information on enzymes used in diagnostic assays and in biotransformation of drugs, which are of pharmaceutical importance, and those have been studied for their biosynthesis using specific microbial strains. These enzymes have their application as an effective and prudent tool in biomedical and pharmaceutical sector, as discussed in following sections.

## 2. Application of enzymes in pharmaceuticals

The enzymatic diagnosis has become a reliable, simple and rapid diagnostic method in medical and health care. It is due to the unique characteristics of enzymes including, their specificity for substrate or chemical compounds, high-catalytic activity under mild conditions of tests. Recent developments in prediction of an illness are the employment of biosensors as potential tools with broader applicability. The enzymes that are well recognised as biological catalysts with specificity and selectivity have been studied for their use as the main component in fabrication of such devices of biosensors [6],[8]. The measurement of activity of specific enzymes in samples collected from patients is used as the method of diagnosis. A particular enzyme is used as a marker for diagnosis of a certain disease in specimens of blood, serum, urine and other body fluids.

Diagnosis by enzyme is a method by measurement of the concentration and changes of certain chemicals in the body to diagnose diseases, which is also done through the changes monitored in activity of the original enzyme in the body fluids. Therefore, this approach of enzymatic diagnostic methods is based on two parameters- firstly, by analysing the changes in the original enzyme activity in the samples taken from patients and secondly, by detecting the changes in some chemicals present in body fluids, which are monitored in tests catalysed by enzymes.

There are several enzymes used as biocatalysts in the diagnostic tests for the detection and also for the treatment of specific diseases [12]–[14], there are some examples presented as below:

1. Glucose oxidase enzyme is widely used to measure glucose level in the diagnosis of diabetes;
2. Cholesterol oxidase enzyme activity can be used to measure the amount of cholesterol present in the blood for diagnosis of condition of hyperlipidemia;
3. Urease enzyme is used to measure the level of urea in blood sample to diagnose liver and kidney lesions;
4. DNA polymerase has its application in testing the state of gene, for normal or any presence of oncogene in the body;
5. Glutaminase enzyme activity is required in the measurement of glutamine content in cerebrospinal fluid for the correct diagnosis of cirrhosis;
6. Trypsin enzyme can be used to dissolve blood clotting, to accelerate the process of wound healing, to remove necrotic tissue as well as in inhibiting the propagation of infecting microflora;
7. L-asparaginase can be used to treat cancer by depriving nutrients needed by the growth of cancer cells;
8. Protease enzyme easily available now in health shops is used to treat digestive disorders, and used by a person for easy digestion of protein-rich diets.

Several other enzymes including, esterase, thrombin, superoxide dismutase, L-asparaginase, lipase, natto-kinase, soybean meal plasmin, etc. have application for their catalytic activity in the medical and healthcare and for the treatment of certain diseases.

Biosensor, as the name states is a device, which uses biological molecules, especially enzymes or antibodies to detect the presence of chemicals in small amount of samples. This analytical device may contain an immobilized biological material (enzyme, antibody, nucleic acid, hormone, organelle or whole cell), which is designed specifically to interact with a specific chemical and produces physical, chemical or electrical signals that can be measured [14],[15]. A biosensor is a self-sufficient integral tool designed which provides precise, quantitative and analytical information. This fabricated device uses a biochemical receptor that is in direct contact with a transduction element.

The biosensor is mainly designed using three main parts: 1. A biological recognition element. 2. A transducer. 3. A signal processing system. Biosensors have become popular because of their accurate, rapid, sensitive and selective detection strategies, which can be used routinely in daily life. Biosensors can be applied for rapid detection of different metabolites (pharmaceuticals) for the diagnosis of various diseases. Those enzymes could be of physiological importance, which are capable of catalyzing a reaction with a chemical or a suspected metabolite under test conditions, these can be employed as a diagnostic entity within a biosensor [11]–[14]. Two diagnostic enzymes discussed in this review are salicylate hydroxylase [13],[16], and dihydro folate reductase [17], which are used for the estimation of specific drugs used in long-term treatment, such as aspirin and methotrexate.

### 2.1. Salicylate hydroxylase

Aspirin (Acetylsalicylic acid), which was first time introduced in medicine was more than a century ago in 1899. This synthetic compound is still one of the most commonly used component in drug formulations prepared for their therapeutic use, as analgesic, antipyretic, and in anti-inflammatory drugs [18]. Aspirin has shown antiplatelet aggregation property, hence, therapeutically this compound acetylsalicylic acid is commonly prescribed to treat cardiovascular problems. Most of the absorbed aspirin enters in the blood circulation after its consumption by patients, in form of salicylate anions. These can be detected in the blood samples, at its highest concentration after two hours the patient has taken all those drug, which contain aspirin in their formulation.

Some patients with certain illnesses are prescribed these drugs for a long-term treatment, therefore, in such cases salicylate levels in plasma samples of patients are measured regularly to avoid their toxic effects [19]–[21]. Though the short-term treatment of aspirin for analgesic/antipyretic purposes are given in low doses, which actually produce considerably low salicylate concentrations, and hence these patients' blood samples do not require monitoring of aspirin levels.

Aspirin, as an anti-inflammatory drug, is given to patients suffering with various forms of arthritis. The blood samples of these patients, in cases of long-term treatment, are regularly monitored to maintain salicylate level within the therapeutic range. Since there is a relatively small difference between therapeutic and toxic dosages, a fast and accurate method is needed to assay the levels of salicylate in blood samples taken from the patients.

Various methods for determining the level of salicylate have been reported in literature. These are a variety of analytical assays including potentiometry analysis using ion-selective electrodes (ISEs) and colorimetric techniques as in the Trinder test [19]. The colorimetric method has several disadvantages and are not very specific [20]. High-pressure liquid chromatography and gas-liquid chromatography, fluorescence and ultraviolet spectrophotometry have also been used [21]–[23], but these assays require time-consuming sample pre-treatment and therefore, such type of analysis are not suitable for use in emergency situations, where a rapid test needs to be performed. Therefore, for a more specific and faster analysis, other assays such as an enzymatic method is used as a solution [24].

Salicylate hydroxylase enzyme (Salicylate 1-monooxigenase, E.C.1.14.13.1.) has been used to determine the salicylate level in pharmaceutical samples and in blood serum [24]. The hydroxylation of the salicylate is catalysed by a biocatalyst, enzyme salicylate hydroxylase, and this biochemical reaction can be used as an assay for the analytical purpose. Salicylate hydroxylase enzyme is required for the hydroxylation and simultaneous decarboxylation of salicylate to catechol.

Following equation explains the enzyme reaction of salicylate with NADH and oxygen, producing catechol, NAD+, carbon di-oxide, and water:

$$\text{Salicylate}+\text{NADH}+{\text{O}}_{\text{2}}+\text{2H}\to \text{Catechol}+\text{NAD}+{\text{H}}_{\text{2}}\text{O}+{\text{CO}}_{\text{2}}$$

In spectrophotometric measurement of enzyme activity, the absorbance decrease of NADH is monitored at 340 nm. The reaction is catalysed by enzyme salicylate hydroxylase, where NADH is used in reaction, as measured by the reduction in absorbance of NADH. The substrate salicylate is converted to catechol, its formation in reaction is indicated by increase in absorption at 276 nm. The stoichiometry between salicylate and NADH has been studied by Zhou et al using salicylate hydroxylase enzyme biosynthesised by *Pseudomonas putida* [13].

#### 2.1.1. Microbial synthesis of salicylate hydroxylase

The interest in synthesis of salicylate hydroxygenase from microbial sources started due to its economical production and recovery of larger yield compared to its extraction from animal tissues. Various microorganisms have been studied for the biosynthesis of this enzyme, including *Trichosporon cutaneum* [25] and *Pseudomonas* species [26]. In our lab a research project was funded by a pharmaceutical company to establish an economical microbial process for the biosynthesis of salicylate hydroxylase. Following a well-designed strategy of enrichment, isolation and screening, a salicylate-hydroxylase producing bacterial strain *Pseudomonas putida* was selected among several isolates. This isolate, with an gradual acclimatisation to survive and grow at toxic concentration of salicylate added in cultivation medium up to 10 g per litre, was studied and selected for the biosynthesis of enzyme [27]. Usually high salicylate concentration above 2 g per litre were found toxic to the growth of microorganisms and have inhibitory effects on salicylate utilising organisms, causing less bacterial cell mass, low biomass yield-coefficients, and overall affecting the enzyme productivity by cells [28]. The biosynthesis process of salicylate enzyme in bacterial cultivation system was optimised for its batch production and then it was gradually scaled up from 500-ml flasks to fermenters with capacity of 5-litres to 80-litres [27].

Since for the recovery of this enzyme, which is not produced extracellularly in the fermentation medium, a large number of bacterial cells were required to release appreciable quantity of cell-bound enzyme for analytical assays. Therefore, to obtain larger volume of fermented medium, cultivation of *Pseudomonas putida* was optimised in a continuous bioreactor system, which yielded a large volume output generating larger amount of bacterial biomass recovered in downstream processing [29]. Continuous cell culture has been reported beneficial to improve low cell densities, and low biomass yield coefficients, which otherwise will result in low enzyme productivity [29],[30].

Pharmaceutically important enzyme salicylate hydroxylase was produced from an isolate *Pseudomonas putida* UUC-1 cultivated in an 80-litre fermenter in 16 h batch process. After downstream processing and purification, each batch fermentation yielded ∼9000 units per 60 litres of working volume in optimized process [27]. The enzyme produced by *P. putida* was purified, partially characterised and used in the construction of a biosensor for the estimation of salicylate (aspirin) in blood samples. Zhou et al. [13] studied use of purified enzyme preparation from an optimised chemostat biosynthesis process employing *P. putida* [16],[29], for its application in the construction of biosensor systems, e.g. carbon-paste electrodes, screen-printed carbon electrodes, and disposable carbon enzyme electrodes.

Although the enzymes are expensive and may increase the cost of the analysis, but the immobilization of enzyme for its repeated use can significantly reduce the cost. The immobilization can be done on solid supports, which are then packed in bioreactors, or closely to an electrode surface. Biosensor for aspirin/salicylate is based on the generation of catechol by the passage of samples with salicylate and nicotinamide adenine dinucleotide (NADH) through an electrode device containing salicylate hydroxylase, which was immobilized in controlled porosity glass beads. The linear range of the disposable carbon enzyme electrode in response to salicylate was achieved to 1.8 mmol/ml [13].

### 2.2. dihydro folate reductase

Methotrexate (MTX), trimethoprim, and pyrimethamine are well known antifolate drugs, and are clinically used as anticancer, antibacterial and antimalarial drugs, respectively, which inhibit dihydrofolate reductase activity. MTX belongs to group of medications termed as antimetabolites. As the MTX treatment slows down the growth of cancer cells and this medication reduces the growth of skin cells to stop forming of scales in condition of psoriasis. Methotrexate is also used in decreasing the activity of the immune system in patients of rheumatoid arthritis [31],[32]. The mechanisms of action MTX in inflammatory arthritis has been recently reviewed in detail [33]. This has been reviewed that despite the use of several agents for the treatment of forms of inflammatory arthritis, therapy with MTX given in low doses is most effective. Therefore, for the treatment of psoriatic arthritis and other forms of inflammatory arthritis MTX is a widely used primary drug.

Dihydrofolate reductase (DHFR) enzyme activity is inhibited by MTX, which prevents the reduction of dihydrobiopterin to tetrahydrobiopterin. That causes aggravation in sensitivity of T cells to apoptosis, as a result immune responses are weakened [34]. MTX can be analysed in serum and plasma samples of patients on long-term treatment taking this drug, DHFR enzyme-inhibition assay is based on principle of MTX inhibiting DHFR by binding to it. The mechanism used in the detection of its concentration, lies in the fact, that the biocatalytic activity of DHFR is inversely proportional to the MTX level in samples. DHFR activity is measured spectrophotometrically at 340 nm as decrease in absorbance of NADPH, which is affected by the presence of MTX in samples.

#### 2.2.1. Microbial synthesis of dihydrofolate reductase

DHFR enzyme (EC1.5.1.3) was initially prepared from animal tissues [35],[36], which was complicated process producing a very low-yield at high cost. Therefore, the biosynthesis of such important biocatalyst was studied using microorganisms particularly from bacterial strains [37],[38]. For higher yield synthesis of DHFR, its production was investigated by six MTX-resistant bacterial strains (PFR-1 to 6) isolated from soil samples. Isolates were subjected to develop resistance for MTX by cultivating them in MTX supplemented medium with the gradual increase in concentration of MTX [17]. Two strains PFR1 and 3 were selected for biosynthesis of DHFR in fermentation process, for their resistance at higher concentration of MTX. High yields up to 4950 Units of enzyme per ml were synthesised by bacterial isolate PFR3 in flask experiments. The production of enzyme was further optimised to obtain an increased yield of 5737 units from 4950 units per litre in scale-up cultivation of bacteria from flasks to 5-litre fermenters [17].

A methotrexate-resistant strain of *Escherichia coli* Type 1 was optimised to produce exceptionally high levels of DHFR enzyme (EC1.5.1.3) within six hours of its fermentation in shake flask-batch process [39]. Further this strain was employed in a chemostat continuous process to obtain a larger yield of enzyme units. A high rate of enzyme biosynthesis was achieved with 10,360 Units per litre in 5-litre fermenters under chemostat culture conditions using a fast growing bacterial strain of *E.coli* [39]. Table 1 summarises published work on two diagnostic enzymes salicylate hydroxylase and dihydrofolate reductase, prepared and characterised using purposely isolated and standard strains of microorganisms, including bacteria and yeasts.

### 2.3. Lipase

The lipase (EC 3.1.1.3) is well-thought-out as a natural catalyst, in humans and animals it is present in the stomach and pancreatic juice as a naturally produced enzyme. The catalytic activity of lipase is required for the digestion of lipids and fats. An active lipolytic system is necessary in helping to maintain function of a healthy gallbladder, in proper digestion of a diet composed of fatty or oily ingredients, regardless from plant or animal source. Lipases belong to the enzyme family of hydrolases, they act on carboxylic ester bonds. They are involved in catalysing the hydrolysis of triglycerides into low density lipoprotein molecules [1],[40].

**Table 1.** Application of microbial Enzymes in diagnostic assays.

Microbial Source	Catalytic Functions	Useful Enzymes	Ref
*Pseudomonas putida UUC-1*	Construction of a biosensor for Aspirin levels	Salicylate hydroxylase	13
Batch culture of *Pseudomonas putida*	Detection of salicylate in body fluids	Salicylate hydroxylase	16
methotrexate-resistant bacterial soil isolates	Antifolate drugs, Methotrexate level in blood samples	Dihydrofolate reductase	17
*Trichosporon cutaneum*	Determining salicylate level in body fluids	hydroxyquinol 1,2-dioxygenase, and Salicylate hydroxylase	25
*Pseudomonas putida ATCC 29351*	electron-transfer properties in reduction of salicylate to Catechol	Salicylate hydroxylase	26
Bacterial Soil isolates	Measurement of salicylate	Salicylate hydroxylase	27
Continuous chemostat culture *P. putida UUC-1*	reduction of salicylate to Catechol	Salicylate hydroxylase	29
*Pseudomanas cepacia* ATCC 29351	Measurement of salicylate in aspirin	Salicylate hydroxylase	30
methotrexate-resistant murine LY5178 cells	Methotrexate level	Dihydrofolate reductase	36
Amethopterin-resistant strain of *Streptococcus faecium*	Level of antifolate drugs	Dihydrofolate reductase	37
Amethopterin-resistant mutant of *Escherichia coli*	Methotrexate level	Dihydrofolate reductase	38
Continuous culture of a methotrexate-resistant *Escherichia coli*	level of antifolate drugs	Dihydrofolate reductase	39

Lipase is the one such widely used and versatile enzyme for the reason that the catalytic mode of lipases could be very different and unique in different reaction medium. For example, at the water-lipid interface lipase have the ability to hydrolyse fats into fatty acids and glycerols, but this reaction could be reversed if lipase working in a non-aqueous medium. This catalytic ability of lipase can be easily exploited for a specific purpose, in catalytic reaction medium with water interface or in absence of water. This possibility of multi-faceted activity of lipase makes it most widely used enzyme for various industrial as well as pharmaceutical applications [41]. The high potential for uses of lipases in health care and medicine are evolving rapidly and currently they are being widely used in treatment. Researchers have explored in intensive studies, lipases can be employed as an important tool in substitution therapy [42]. In some patients lipase enzyme deficiency occurs during illness and poor health conditions, then the required doses of lipase are administered externally, to compensate the low lipase level in patients. Under normal metabolic condition occurring in healthy human beings, lipase are playing their role in breaking down fats present in diet. As a result fat content is digested and absorbed in the intestine, but in the compromised health condition the deficiency of lipases leads to malabsorption of fats and also other fat-soluble nutrient and vitamins [41],[42].

Lipases therapy can be used in the treatment of Alzheimer's disease, atherosclerosis cystic fibrosis, and for prevention and therapy for cancer. They have potential in their application as a diagnostic tool for the detection of their presence or varied levels in blood, which can indicate the detection of certain disease or infection [42]. Obesity is a serious health issue of concern around the world, the state of obesity causes metabolic disorders. The practical pharmacological approach considered in the treatment of obesity is by inhibiting the activity of digestive lipase, which will reduce fat absorption due to its non-assimilation in body of patient or a person with condition of obesity [43].

Lipase enzymes can be applied as biocatalysts to perform the conversion or transformations of highly regio- and stereo-selectivity under milder conditions, compared to chemical reactions. For the wider utility in several industries, lipases from microbial sources have been studied, as they are used as important tool for their catalytic activity of biotransformation [40],[44]. The Lipase is significantly important in pharmaceutical and agro-chemical industry therefore, stereo-selectivity of lipase has been exploited for the preparation of single enantiomers from racemic mixtures, for the desired activity of biotransformation.

The work has been published related to enzymatic bio-transformation of chiral compounds for application in pharmaceutical industry [45],[46]. Lipase enzyme preparation from selected yeast strains has been used in the biotransformation studies by Muralidhar et al, for the racemic resolution of RS-Baclofen [47], and for the resolution of (RS)-proglumide [48].

#### 2.3.1. Microbial synthesis of lipase

The activity of these enzymes are adequately stable and the supply of enzyme can be obtained from *in vivo* synthesis sources including, animals and plants. But to get better and higher yields of lipase, this can be *in vitro* synthesized in laboratory employing selected microorganisms. Therefore, for large-scale requirement of lipase in industries for commercial use, lipases significantly synthesized by microbial strains are being used [1],[40]. Extensive work has been published on various substrates and methods studied for the biosynthesis of lipase employing microbial strains [1],[40],[44]. Lipase has been synthesized using different microbial strains (Table 2).

The synthesis of lipase by microorganisms mainly depends on culture conditions used in the fermentation [49]. It is important to select a suitable process for biosynthesis depending on type of carbon sources being used [50]. Researchers have studied different states of fermentation process, including batch, fed-batch to increase the production rate of enzyme [51]. For the synthesis of higher yield of lipase enzyme, a yeast strain of *Candida cylindraceae* NRRL Y-17506 was employed using two types of carbon sources olive oil, glucose and combinations of glucose/olive oil in submerged fermentation. A response surface approach was adopted for the comparison of lipase production, with various medium composition using glucose/olive oil, malt extract, yeast extract, peptone and tween 80 [52]. An effective assay for lipase enzyme activity has been recommended by Horiuti et al, which is a titrimetric method by substrate emulsification using olive oil as a substrate in reaction mixture and Tris-HCl buffer. One lipase unit is calculated as the release of one micro-equivalent of fatty acid per minute under standard assay condition of 27 °C at pH 8.0 [53].

A novel solvent stable lipase enzyme has been synthesised by a bacterial culture *Pseudomonas* and characterised for its stability in solvent medium [54]. A purified preparation of lipase produced by fungal strain *Aspergillus fumigatus* has been characterised for its suitability in selected applications [55]. Lipases have established their use in the construction of biosensors [56], due to their wide substrate specificity in the conversion of triglycerides and other esterified substrates. Moreover, now lipases are commercially available for several applications, including medical diagnosis and bioassays [57]–[59].

**Table 2.** Application of microbial enzymes in biotransformation.

Catalytic Functions	Useful Enzymes	Microbial Source	Ref
Industrial uses for lipolytic activity	Lipase	Several microbial strains	40
racemic resolutions	Lipase	Several yeast strains	44
Captopril synthesis	Esterase	Bacterial strains	45
Stereo-selectivity resolution	Lipase	Several microbial strains	46
Racemic resolution of RS-Baclofen	Lipase	*Candida cylindraceae*	47
Resolution of (RS)-Proglumide	Lipase	*Candida cylindraceae*	48
Resolution of antibiotics	Acidic Lipase	*Rhizopus arrhizus*	49
Industrial uses	Lipase	*Candida rugosa*	50
Industrial uses	*Pseudomonas fluorescence*-lipase	Recombinant *Escherichia coli* in Fed-batch cultivation	51
Stereo-selectivity resolution	Lipase	*Candida cylindracea*	52
Industrial uses	novel solvent stable lipase	*Pseudomonas reinekei*	54
Industrial uses	Purified lipase	*Aspergillus fumigatus*	55

## 3. Conclusion and future perspectives

With a change in lifestyle and an increasing ageing population in many countries, diseases and health-disorders are also increasing. Therefore, accurate test methods results are needed to deal with the increasing number of health issues, and for the timely treatment of a particular diagnosed condition. Different enzymes, diagnostic kits and biosensors are being evaluated for monitoring and diagnosis of the types of suspected ailments. Diagnostic tests have been widely used to detect and quantify different biomarkers to attain the critical information about health care, which is an important step in making accurate medical decisions. The major challenges with use of enzymes for diagnostic purposes are their yield, shelf life and the stability, if these enzymes were extracted from non-microbiological sources, such as animal tissues.

Several diagnostic methods used in pathology labs require costly reagents, equipment, and time for analysis of samples, as well as the availability of trained pathologists and technical staff to operate the process. Hence, there is a focus on research and development of tools that are cost-effective, stable and simple-to-operate. The enzyme-based detection of various health disorders have been investigated and in some cases it is fully established; but high costs are involved in enzyme production, purification, their shelf life and storage conditions. For an economical production of enzymes by a humane technique (avoiding their extraction from animal tissues), the use of microbial fermentation technology is a preferred option. Enzymes synthesized by selected microbial strains can be purified for high specific activities and characterized for their stability. Such bioactive enzyme preparations, either in a free-state or in an immobilized-state, can be utilised for diagnostic purposes with specificity and accuracy.
